# Is combining human service work with family caregiving associated with additional odds of emotional exhaustion and sickness absence? A cross-sectional study based on a Swedish cohort

**DOI:** 10.1007/s00420-019-01461-0

**Published:** 2019-07-25

**Authors:** Emma Drake, Susanna Toivanen, Constanze Leineweber, Anna Nyberg

**Affiliations:** 1 Stress Research Institute, Stockholm University, Stockholm, Sweden; 2 Department of Public Health Sciences, Stockholm University, Stockholm, Sweden; 3 School of Health, Care, and Social Welfare, Mälardalen University, Västerås, Sweden

**Keywords:** Human service work, Informal caregiving, Family caregiving, Double duty caregiving, Emotional exhaustion, Sickness absence

## Abstract

**Purpose:**

The aim of the study is to examine to what extent human service work and family caregiving is associated with emotional exhaustion and sickness absence, and to what extent combining human service work and family caregiving is associated with additional odds.

**Methods:**

Data were derived from participants in paid work from the Swedish Longitudinal Occupational Survey of Health, year 2016 (*n* = 11 951). Logistic regression analyses were performed and odds ratios and 95% confidence intervals estimated for the association between human service work and family caregiving, respectively, as well as combinations of the two on one hand, and emotional exhaustion and self-reported sickness absence on the other hand. Interaction between human service work and family caregiving was assessed as departure from additivity with Rothman’s synergy index.

**Results:**

Human service work was not associated with higher odds of emotional exhaustion, but with higher odds of sickness absence. Providing childcare was associated with higher odds of emotional exhaustion, but lower odds of sickness absence, and caring for a relative was associated with higher odds of both emotional exhaustion and sickness absence. There was no indication of an additive interaction between human service work and family caregiving in relation to neither emotional exhaustion nor sickness absence.

**Conclusions:**

We did not find support for the common assumption that long hours providing service and care for others by combining human service work with family caregiving can explain the higher risk of sickness absence or emotional exhaustion among employees in human service occupations.

## Background

In Sweden, human service occupations employ over 800,000 individuals in healthcare, elderly care and education and employees in them constitute a large part of the working population (Swedish Social Insurance Agency 2014). Employees working with human service have been found to have higher risk of stress-related disorders (Wieclaw et al. 2006), more sickness absence due to mental disorders (Swedish Social Insurance Agency 2014), more doctor-certified sickness absence (Aagestad et al. 2016) and higher risk of antidepressent use (Buscariolli et al. 2018) than other employees. In the present study we define human service work as “direct contact with patients or other care-intensive persons, alternatively with children and adolescents whose educational development or care one is responsible for in one’s work”.

Various possible explanations for the higher risk of sickness absence for those in human service work have been suggested, but there is, to date, no consensus about which factors to target with interventions to improve mental health and prevent new cases of sickness absence in these professional groups. Previous studies have shown that a poorer psychosocial work environment in human service occupations contributes to higher risks of burnout and sickness absence in these professions (Aagestad et al. 2016; Aronsson et al. 2019; Bria et al. 2012). The emotional and interpersonal stressors from providing service and care to other people have been suggested and reported to be one of the most influential factors (Aronsson et al. 2019; Bria et al. 2012; Maslach et al. 2001). Another factor that could explain the high risks of sickness absence among employees working in human service occupations may be found in the high total number of working hours in provision of service and caregiving arising from combining paid and unpaid work (AFA Försäkring 2015). Thus, in accordance with the role strain hypothesis (Goode 1960), suggesting that multiple roles in life create increased stress and demands on time, energy, and psychological resources, combining emotionally demanding work with those in need at home and at work may have negative health consequences.

Caregiving in family life can consist both of caregiving of children as a normal part of child rearing, and of caregiving for a sick, disabled or elderly relative, often referred to as informal caregiving (Mortensen et al. 2017). Time in childcare activities has been found to contribute to emotional exhaustion and sickness absence (Bekker et al. 2005) and sickness absence has been found to be higher among women with children than among women without (Floderus et al. 2012), particularly among women who report work–family conflict (Voss et al. 2008a). In Sweden, the risk of entering a sickness period due to mental disorders has been found to increase markedly among parents 2 years after the second child is born, at the point in time when both parents usually return to work after parental leave (Swedish Social Insurance Agency 2014), indicating a work–family conflict. However, other studies show that the association between having children and sickness absence is weak (Mastekaasa 2000). Caring for a relative, on the other hand, has in previous studies been shown to increase allostatic load (Dich et al. 2015), poor sleep (Sacco et al. 2017), poor self-rated health (Legg et al. 2013), and high risk of sickness absence for women (Mortensen et al. 2017). Longer hours of caring for a relative have furthermore been found to influence mental ill-health among nurses (Cannuscio et al. 2002). Previous research has found the so called double-duty care givers (Ward-Griffin et al. 2015) to report increased stress, psychological distress (DePasquale et al. 2016) and emotional exhaustion (Boumans and Dorant 2014). In this literature a distinction is made between those who work with formal caregiving and provide care for children in their spare time, *double*-*duty child caregivers*, and those who care for relatives or other dependent adults, *double*-*duty adult caregivers* (Hausler et al. 2017). The research on double-duty caregivers is still scarce (DePasquale et al. 2016; Hausler et al. 2017) and has, to date, mostly focused on healthcare personnel. To the best of our knowledge, there are no studies in which employees with and without caregiving professions are compared with regards to associations between hours of caregiving and mental ill-health and sickness absence. The present study contributes by filling this gap in a sample including a wide range of human service occupations.

The aim of the study is to examine to what extent human service work and family caregiving are associated with emotional exhaustion and sickness absence, and to what extent combining human service work and family caregiving is associated with additional odds of these outcomes. The specific research questions are:

1. Is there an association between hours in human service work and family (caring for children or caring for a relative) caregiving on one hand and emotional exhaustion or sickness absence on the other hand?
2. Is there an additive interaction between human service work and family caregiving with regards to odds for emotional exhaustion and sickness absence?

## Methods

### Data material and study sample

**Fig. 1:**
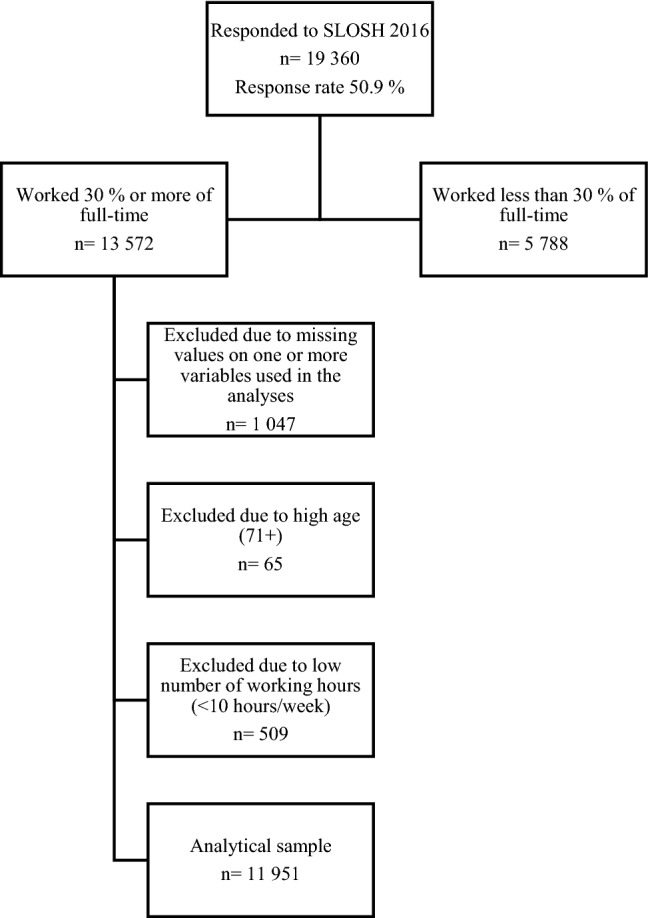
Flow chart illustrating how the analytical sample was reached from the sample of responders to SLOSH 2016

Data were drawn from the Swedish Longitudinal Occupational Survey of Health (SLOSH), an approximately nationally representative sample of Sweden’s working population. The present study is based on the data collection from 2016 (*n* = 19 360, response rate 50.9%). The first wave of SLOSH was carried out in 2006 as a follow-up to the Swedish Work Environment Survey (SWES) 2003, in turn sampled from the Labour Force Survey (LFS). Further SWES cohorts have been added and today SLOSH consists of SWES participants from 2003–2011. The responders to SLOSH are invited to answer a self-completion questionnaire in two versions, one for those who worked 30% or more of full time the past 3 months and another for those who worked less or not at all. Informed consent was obtained from all individual participants included in the study. More information on SLOSH can be found in the cohort profile (Magnusson Hanson et al. 2018). Only participants who had worked 30% or more over the past 3 months and answered the questionnaire in year 2016 were included (*n* = 13,572). Furthermore, individuals with missing values on one or more of the items used in the analyses (*n* = 1047) and individuals who reported working less than 10 h per week (*n* = 509) were excluded. Also participants 71 years and older were excluded (*n* = 65) due to low participation in the working force in this age group. The final study sample consisted of 11,951 individuals (Fig. 1).

### Exposure variables

Human service work was measured with the question “Do you have direct contact with patients or other care-intensive persons, alternatively with children and adolescents whose educational development or care you are responsible for in your work?” The response alternatives were “No”, “Yes, less than half of my worktime”, and “Yes, half of my worktime or more”. For the first research question all three response alternatives were analysed and for the second one the answers were dichotomized into *Human service work* (“Yes, less than half of my worktime”, and “Yes, half of my worktime or more”) and *No human service work* (“No”). A question used to measure several household activities was used to measure time caring for children and for relatives: “If you think about an ordinary week of 7 days, how many hours do you spend on the following activities?” Time caring for children (pick-up, leave, homework, care, and supervision) and caring for relatives were measured with the response alternatives 0, 1–5, 6–10, 11–15, or > 15 h/week. For the first research question, all response alternatives were analysed, but for the second one they were dichotomized into *short* (< 11 h) or *long* (≥ 11) hours per week in childcare and into *not caring for relatives* (0 h/week) and *caring for relatives* (≥ 1 h/week). These cutoffs were based on to what extent the number of hours of care can be considered as demanding in an everyday life, the number of individuals reporting these numbers of hours, as well as the evidence regarding associations with mental health outcomes. For example, up to 10 h of childcare per week may be considered as a normal part of life and an activity that may be rewarding. On the other hand, relatively few people care for a relative and because such responsibilities more consistently have been reported to be associated with mental health outcomes, we placed the cutoff between providing and not providing care for a relative.

### Outcome variables

Emotional exhaustion was measured with eight items from the Shirom-Melamed Burnout Questionnaire (SMBQ) forming the subscale of emotional/physical fatigue. Example items are “I feel tired” and “I’m fed up”. The answers were scored on a 7-point scale from “almost never” to “almost always”. An average index score was computed and dichotomised with the cutoff point at ≥4.4 (Lundgren-Nilsson et al. 2012). The Cronbach’s alpha for the scale was 0.92, indicating good internal consistency. Sickness absence was measured by the question: roughly how many days in total have you been on sick leave during the past 12 months? The response alternatives were “Not at all”, “1–7”, “8–30”, “31–90” or “91 days or more”. Sickness absence was used as a binary variable with the categories “sickness absence” (31 days or more) and “reference group” (0–30 days of sickness absence). In line with previous studies (Aronsson et al. 2019; Bergstrom et al. 2009; Leineweber et al. 2017), we chose a cutoff of 31 days or more to indicate sickness absence, aiming to reflect rather recurrent, long-lasting, and/or severe health problems.

### Covariates

Gender, age, education and civil status were all based on registry data. The participants were categorized as either man or woman. Age was adjusted for in the categories < 30, 31–40, 41–50, 51–60, and 61–70. Civil status was measured as married/cohabiting or single. Education consisted of the five categories compulsory education, 2-years upper secondary education/vocational training, 3 or 4 years upper secondary, university or equivalent shorter than 3 years, and university or equivalent 3 years or longer. Working hours were self-reported in the categories: 10–19, 20–29, 30–35, 36–40, 41–45, 46–50, 51–55, 56–60 and > 60 h per week. The chosen covariates were included based on earlier research regarding their associations with the outcomes (Maslach et al. 2001; Swedish Social Insurance Agency 2014) and the different distributions of the background variables between individuals with and without human service work. Single mothers have in earlier studies been found to have higher risk of sickness absence and this contributed to adding civil status as a covariate (Floderus et al. 2012; Voss et al. 2008a).

### Statistical analyses

The descriptive statistics are presented stratified by human service workers and other workers. Chi^2^ tests were used to estimate the statistical significance of differences in the study variables. Multiple logistic regression analyses were performed to calculate odds ratios and 95% confidence intervals for human service work and family (caring for children and caring for relative) caregiving, respectively, for the two outcomes emotional exhaustion and sickness absence. Three different models were built for each independent variable, where the first one was unadjusted, the second one adjusted for age, gender, education and civil status and the third one adjusted additionally for working hours. In the second step human service work (yes/no) and family caregiving were combined to form four different exposure groups for caring for children (short/long hours) and caring for relative (yes/no), respectively. Odds of emotional exhaustion and sickness absence were estimated with the group not exposed to human service work or family caregiving as the reference category. The models were built using the same procedure as above, with the first model unadjusted, the second model adjusted for gender, age, education and civil status, and the third one adjusted additionally for working hours. Interaction between human service work and family caregiving was measured as departure from additivity with Rothman’s synergy index (Rothman 1976). A synergy index higher than one indicates a positive interaction and an index lower than one a negative interaction. The analyses were calculated using the procedure proposed by Andersson et al. (2005) (Ahlbom and Alfredsson 2005). All statistical analyses were performed in IBM SPSS Statistics Version 25.

## Results

Table 1 shows frequencies and percentages of all variables in the study stratified by those who worked with human service and those who did not. Of the total sample, 30% worked with human service and 70% did not. Among employees that worked with human service, 83% were women and 17% were men and among those in other occupations there were 45% women and 55% men. Human service workers were more likely to be older, have a university education, work less than 35 h per week, and spend less time caring for children and more time caring for a relative. There was a higher prevalence of the outcomes emotional exhaustion and sickness absence among human service workers. Drop-out analysis showed that excluded participants (due to missing values) more often were women, had lower education, higher age and worked with human service compared with the analytical sample.

**Table 1:** Frequencies and percentages for covariates, caring for children, caring for relative, emotional exhaustion and sickness absence stratified between individuals with and without human service work

	Human service workers (*n* = 3616)	Other workers (*n* = 8335)
	*n* (%)	*n* (%)
Age of participants		
30 and under	67 (1.9)	189 (2.3)
31–40	428 (11.8)	1122 (13.5)
41–50	901 (24.9)	2452 (29.4)
51–60	1413 (39.1)	3020 (36.2)
61–70	807 (22.3)	1552 (18.6)
Women	2984 (82.5)	3716 (44.6)
Education		
Compulsory	256 (7.1)	776 (9.3)
2-year upper secondary	505 (14.0)	1966 (23.6)
3–4 year upper secondary	519 (14.4)	2333 (28.0)
University less than 3 years	677 (18.7)	1042 (12.5)
University 3 years or more	1659 (45.9)	2218 (26.6)
Civil status		
Single	746 (20.6)	1655 (19.9)
Married/cohabiting	2870 (79.4)	6680 (80.1)
Working hours		
Less than 35 h/week	989 (27.4)	1225 (14.7)
36–45 h/week	2131 (58.9)	5506 (66.1)
46 h or more/week	496 (13.7)	1604 (19.2)
Caring for children		
0 h/week	2401 (66.4)	5258 (63.1)
1–5 h/week	683 (18.9)	1801 (21.6)
6–10 h/week	281 (7.8)	733 (8.8)
11–15 h/week	84 (2.3)	251 (3.0)
> 15 h/week	167 (4.6)	292 (3.5)
Caring for relative		
0 h/week	3040 (84.1)	7363 (88.3)
1–5 h/week	486 (13.4)	841 (10.1)
6–10 h/week	57 (1.6)	78 (0.9)
11–15 h/week	14 (0.4)	20 (0.2)
> 15 h/week	19 (0.5)	33 (0.4)
Outcomes		
Emotional exhaustion	383 (10.6)	752 (9.0)
Sickness absence	301 (8.3)	460 (5.5)

### Human service work, family caregiving and emotional exhaustion

As shown in Table 2, individuals who worked with human service more than 50% of their worktime had higher odds of emotional exhaustion (OR = 1.20, 95% CI 1.04–1.38) in the unadjusted model compared with individuals who did not work with human service. The association did, however, not remain significant after adjustment for covariates in models 2 and 3. With regard to family caregiving, participants who spent 6 h or more per week caring for children had higher odds of emotional exhaustion compared with those who spent 0 h per week in the unadjusted model. Participants caring for children 11–15 h per week were the only group with significantly higher odds of emotional exhaustion compared with those who spent 0 h per week caring for children after adjustment for covariates in models 2 and 3. Caring for a relative was associated with significantly higher odds of emotional exhaustion in all time span categories in the unadjusted model. The associations remained significant after adjustments in models 2 and 3. The odds of emotional exhaustion increased for each category of increasing hours spent caring for a relative, indicating a dose–response relationship.

**Table 2:** Odds ratios (OR) with 95% confidence intervals (CI) of emotional exhaustion for time in human service work and family caregiving respectively (*n* = 11,951)

		Emotional exhaustion		
	*N*	Model^1^ OR (95% CI)	Model^2^ OR (95% CI)	Model^3^ OR (95% CI)
*Human service work*				
No (ref)	8335	1	1	1
< 50%	686	1.18 (0.92–1.53)	1.11 (0.85–1.44)	1.09 (0.84–1.41)
> 50%	2930	1.20 (1.04–1.38)	1.06 (0.91–1.23)	1.05 (0.90–1.23)
*Family caregiving*				
Caring for children				
0 h/week (ref)	7659	1	1	1
1–5 h/week	2484	1.04 (0.89–1.22)	1.03 (0.86–1.23)	1.03 (0.86–1.22)
6–10 h/week	1014	1.34 (1.09–1.65)	1.25 (0.99–1.57)	1.23 (0.98–1.55)
11–15 h/week	335	1.93 (1.43–2.62)	1.78 (1.29–2.47)	1.78 (1.29–2.47)
> 15 h/week	459	1.49 (1.12–1.98)	1.24 (0.90–1.69)	1.24 (0.90–1.70)
Caring for relative				
0 h/week (ref)	10,403	1	1	1
1–5 h/week	1327	1.32 (1.10–1.58)	1.37 (1.13–1.65)	1.36 (1.13–1.64)
6–10 h/week	135	1.97 (1.24–3.12)	2.01 (1.26–3.22)	2.05 (1.28–3.28)
11–15 h/week	34	2.62 (1.14–6.03)	2.56 (1.09–6.01)	2.40 (1.02–5.63)
> 15 h/week	52	4.49 (2.48–8.12)	4.42 (2.42–8.09)	4.37 (2.38–8.03)

Model^1^: Unadjusted

Model^2^: Adjusted for gender, age, education and civil status

Model^3^: Model^2^ + working hours

### Human service work, family caregiving, and sickness absence

As shown in Table 3, employees working with human service both less than 50% and 50% or more of their worktime had significantly higher odds of sickness absence in the unadjusted model compared with employees not working with human service. The associations were still significant after adjustment for gender, age, education, and civil status. However, only the group working less than 50% with human service had significantly higher odds of sickness absence (OR = 1.35, 95% CI 1.00–1.80) after additional adjustment for working hours. Hours caring for children were negatively associated with sickness absence, such that individuals who spent 1–10 h per week on childcare had significantly lower odds of sickness absence than those spending 0 h in caring for children in the unadjusted model. Only individuals caring for children 1–5 h per week had significantly lower odds of sickness absence after full adjustment (OR = 0.76, 95% CI = 0.61–0.95). Individuals who spent more than 15 h per week caring for a relative had significantly higher odds of sickness absence in all three models compared with individuals who did not care for a relative. The odds ratio of sickness absence was 3.14 (95% CI 1.49–6.59) for participants who did more than 15 h of caregiving per week in the fully adjusted model.

**Table 3:** Odds ratios (OR) with 95% confidence intervals (CI) of sickness absence for time in human service work and family caregiving, respectively (*n* = 11,951)

		Sickness absence		
	*N*	Model^1^ OR (95% CI)	Model^2^ OR (95% CI)	Model^3^ OR (95% CI)
*Human service work*				
No (ref)	8335	1	1	1
< 50%	686	1.61 (1.21–2.14)	1.40 (1.05–1.87)	1.35 (1.00–1.80)
> 50%	2930	1.54 (1.31–1.81)	1.26 (1.06–1.50)	1.12 (0.94–1.34)
*Family caregiving*				
Caring for children				
0 h/week (ref)	7659	1	1	1
1–5 h/week	2484	0.68 (0.56–0.84)	0.77 (0.62–0.96)	0.76 (0.61–0.95)
6–10 h/week	1014	0.70 (0.52–0.94)	0.82 (0.59–1.13)	0.79 (0.57–1.09)
11–15 h/week	335	0.88 (0.56–1.39)	1.07 (0.67–1.72)	1.01 (0.62–1.62)
> 15 h/week	459	0.86 (0.58–1.27)	1.01 (0.66–1.54)	0.85 (0.55–1.32)
Caring for relative				
0 h/week (ref)	10,403	1	1	1
1–5 h/week	1327	1.25 (1.00–1.56)	1.10 (0.88–1.38)	1.14 (0.91–1.42)
6–10 h/week	135	1.50 (0.82–2.72)	1.21 (0.66–2.21)	1.30 (0.71–2.38)
11–15 h/week	34	1.48 (0.45–4.87)	1.34 (0.41–4.43)	1.22 (0.36–4.08)
> 15 h/week	52	3.21 (1.56–6.61)	2.84 (1.37–5.89)	3.14 (1.49–6.59)

Model^1^: Unadjusted

Model^2^: Adjusted for gender, age, education and civil status

Model^3^: Model^2^ + working hours

### Combinations of human service work and family caregiving and emotional exhaustion

As shown in Table 4 employees who spent 11 h or more caring for children per week had higher odds of emotional exhaustion in the fully adjusted model (OR = 1.50, 95% CI 1.04–2.16 and OR = 1.36, 95% CI 1.03–1.79, respectively) when compared to the reference category regardless of if they worked with human service or not. The same pattern was found regarding caring for a relative, where individuals caring for a relative had higher odds of emotional exhaustion in the fully adjusted model (OR = 1.33, 95% CI = 1.01–1.76 and OR = 1.75, 95% CI = 1.42–2.16, respectively) regardless of type of occupation. The analyses of departure from additivity showed no indication of an interaction between human service work and caring for children. There was a tendency towards a negative interaction between human service work and caring for a relative (synergy index 0.38, 95% CI 0.13–1.16), however not statistically significant.

**Table 4:** Odds ratios (OR) with 95% confidence intervals (CI) of emotional exhaustion for combinations of human service work (HSW) and family caregiving (*n* = 11,951)

		Emotional exhaustion		
	*N*	Model^1^ OR (95% CI)	Model^2^ OR (95% CI)	Model^3^ OR (95% CI)
Human service work and caring for children				
No HSW & Childcare < 11 h/week (ref)	7792	1	1	1
No HSW & Childcare ≥ 11 h/week	543	1.57 (1.21–2.04)	1.35 (1.02–1.78)	1.36 (1.03–1.79)
HSW & Childcare < 11 h/week	3365	1.19 (1.03–1.36)	1.06 (0.92–1.23)	1.06 (0.91–1.23)
HSW & Childcare ≥ 11 h/week	251	1.98 (1.40–2.80)	1.51 (1.05–2.18)	1.50 (1.04–2.16)
Human service work and caring for relative				
No HSW & no caregiving (ref)	7363	1	1	1
No HSW & caregiving ≥1 h/week	972	1.69 (1.38–2.07)	1.76 (1.43–2.16)	1.75 (1.42–2.16)
HSW & no caregiving	3040	1.26 (1.09–1.45)	1.13 (0.97–1.32)	1.12 (0.96–1.31)
HSW & caregiving ≥ 1 h/week	576	1.43 (1.09–1.87)	1.35 (1.02–1.79)	1.33 (1.01–1.76)

Synergy index HSW/caring for children (model^3^): 1.19 (95 % CI 0.31–4.63)

Synergy index HSW/caring for relative (model3): 0.38 (95 % CI 0.13–1.16)

Model^1^: Unadjusted

Model^2^: Adjusted for gender, age, education and civil status

Model^3^: Model^2^ + working hours

### Combinations of human service work and family caregiving and sickness absence

As shown in Table 5 employees who worked with human service and who spent either long or short hours caring for children per week had higher odds of sickness absence in the unadjusted model compared with the reference category, and the associations remained significant after adjustment for gender, age, education and civil status, but not with the introduction of working hours. For combinations with caring for a relative, only employees with human service work without caring for a relative had significantly higher odds of sickness absence compared with the reference category after adjustment for gender, age, education and civil status. The analyses of departure from additivity showed no indication of an interaction between human service work and caring for children or caring for a relative.

**Table 5:** Odds ratios (OR) with 95% confidence intervals (CI) of sickness absence for combinations of human service work (HSW) and family caregiving (*n* = 11,951)

		Sickness absence		
	*N*	Model^1^ OR (95% CI)	Model^2^ OR (95% CI)	Model^3^ OR (95% CI)
Human service work and caring for children				
No HSW & Childcare < 11 h/week (ref)	7792	1	1	1
No HSW & Childcare ≥ 11 h/week	543	0.89 (0.60–1.33)	1.07 (0.71–1.62)	0.97 (0.64–1.47)
HSW & Childcare < 11 h/week	3365	1.54 (1.31–1.80)	1.27 (1.07–1.50)	1.14 (0.96–1.36)
HSW & Childcare ≥ 11 h/week	251	1.63 (1.04–2.56)	1.71 (1.07–2.74)	1.38 (0.86–2.23)
Human service work and caring for relative				
No HSW & no caregiving (ref)	7363	1	1	1
No HSW & caregiving ≥1 h/week	972	1.44 (1.11–1.87)	1.28 (0.98–1.66)	1.31 (1.00–1.71)
HSW & no caregiving	3040	1.60 (1.36–1.89)	1.33 (1.11–1.59)	1.19 (1.00–1.43)
HSW & caregiving ≥ 1 h/week	576	1.78 (1.32–2.42)	1.35 (0.99–1.85)	1.28 (0.94–1.76)

Synergy index HSW/caring for children (model^3^): 3.47 (95% CI 0.04–278.49)

Synergiindex HSW/caring for relative (model^3^): 0.57 (95% CI 0.13–2.55)

Model^1^: Unadjusted

Model^2^: Adjusted for gender, age, education and civil status

Model^3^: Model^2^ + working hours

## Discussion

In the present study we investigated to what extent human service work and family caregiving were associated with emotional exhaustion and sickness absence, and if combining human service work and family caregiving was associated with additional odds of these outcomes. Results showed that human service work was not associated with increased odds of emotional exhaustion, but with increased odds of sickness absence. Family caregiving, both caring for children and caring for a relative, was associated with increased odds of emotional exhaustion. Long hours caring for a relative was associated with higher odds of sickness absence, while hours in childcare were associated with lower odds of sickness absence. There was no support for the notion that combining human service work and family caregiving would be associated with additional odds of emotional exhaustion or sickness absence.

### Human service work

The present results confirm previous findings showing that work with human service is associated with higher odds of sickness absence (Aagestad et al. 2016; Aronsson et al. 2019; Swedish Social Insurance Agency 2014). Unexpectedly, providing human service less than half of the worktime, but not half of the worktime or more, was associated with higher odds of sickness absence in the fully adjusted model. This contradicts the notion that longer hours in human service, with assumed exposure to high emotional demands, would be associated with higher odds of sickness absence. The result is particularly unexpected because high emotional demands have been found to be one of the core factors explaining differences in sickness absence between employees in caregiving occupations and employees in other occupations (Aronsson et al. 2019). The results suggest that other factors may be more important for sickness absence in caregiving professions than the direct contact with clients, such as for example poor work time control, organisational injustice, or emotional demands associated with other relationships at work than relationships with clients (Aagestad et al. 2016; Aronsson et al. 2019). Also, based on our data, we cannot rule out the possibility that there has been a selection of individuals into work tasks requiring less contact with clients based on for example health status or particular life circumstances.

### Family caregiving

#### Caring for children

The result showing that long hours caring for children were associated with higher odds of emotional exhaustion are in line with previous results showing that hours caring for children contributed to emotional exhaustion among male and female nurses in the Netherlands (Bekker et al. 2005). However, the Dutch researchers also found a positive association with sickness absence, whereas the present results show a negative association. Parenthood has previously been linked to higher risk of sickness absence (Floderus et al. 2012; Swedish Social Insurance Agency 2014), although some studies report weak associations (Mastekaasa 2000) mostly confined to single mothers (Floderus et al. 2012; Voss et al. 2008a) and mothers reporting work–family conflict (Voss et al. 2008a) who are at higher risk. There may be several reasons for the contradicting result of the present study. First, we analyse mothers and fathers together, thus do not focus on mothers explicitly. We furthermore investigate hours of childcare rather than having children or not, and measure self-reported all-cause sickness absence, whereas for example the data from the Swedish Social Insurance Agency (2014) where positive associations were found, are register-based sickness absence periods exceeding 14 days due to mental disorders. The negative association between caring for children and sickness absence found in this study supports the *role accumulation theory* (Sieber 1974), proposing for example that caring for a child may provide sources of personal satisfaction and self-esteem, possibly associated with lower odds of sickness absence.

#### Caring for a relative

The present findings suggest a linear relationship between hours caring for a relative and emotional exhaustion. These results support the notion that emotional exhaustion can have causes not only in working life but also in private life (Bianchi et al. 2017) and are in line with earlier publications in which caring for a relative has been found to be associated with high allostatic load and poor self-reported health (Dich et al. 2015; Legg et al. 2013). Individuals who care for a sick or disabled close relative, such as a child or a partner, usually give the most extensive help (Szebehely 2014), which may correspond to our group spending 15 h or more per week, and explain the substantial association with poor mental health. Caring for children and caring for a relative appear to be distinct phenomena with different health implications, where particularly individuals caring for a relative appear to be at high risk of ill-health.

### Combining human service work and family caregiving

#### Human service work and caring for children

There was no indication of an additive interaction between human service work and caring for children in relation to any of our outcomes. Long hours of caring for children were associated with higher odds of emotional exhaustion irrespective of human service work. This contradicts previous research on double-duty caregivers in which working in healthcare and providing care for children was not shown to be associated with perceived stress (DePasquale et al. 2016) or emotional exhaustion (Hausler et al. 2017), but in these studies occupational types were not compared—only health care professionals were included. No support for the hypothesis that longer total hours in service and caregiving would be associated with higher odds of having emotional exhaustion was found. Interestingly, employees with long hours of caring for children had increased odds of emotional exhaustion, whereas only employees with human service work, irrespective of number of hours providing childcare, had increased odds of sickness absence. One could speculate that when level of emotional exhaustion is comparable, this health condition may more often lead to sickness absence among individuals who work with human service. This may be due to lack of work flexibility and work time control (Irvine 2011), or a poorer psychosocial work environment (Aagestad et al. 2016; Aronsson et al. 2019), making work harder to manage when emotional exhaustion is experienced. It has also been suggested that employees working under poor conditions may use short-term sickness absence as a strategy for recuperation to not get ill (Colquitt et al. 2001; Duijts et al. 2007).

#### Human service work and caring for relative

A similar result was found for combining human service work and caring for a close relative—no additive interaction was observed. None of the groups differed significantly in terms of odds for sickness absence after adjustment for all control variables. Regarding emotional exhaustion, caring for a relative was associated with higher odds of the outcome both among individuals working with human service and among those who did not. Thus, no support for higher odds of sickness absence and emotional exhaustion associated with combining human service work and caring for a relative was found. In contrast the result from the interaction analysis showed a tendency of somewhat higher odds of emotional exhaustion among individuals with no human service work who cared for a relative compared with individuals working formally with caregiving. In a previous study nurses reported that their knowledge of the health care system was beneficial as informal caregivers, for example the ability to access information and resources (Ward-Griffin et al. 2015). The lack of such knowledge, experience, and access to information may be one explanation of this tendency. Combining human service work and caring for a relative has in earlier studies been associated with perceived stress (DePasquale et al. 2016) and emotional exhaustion (Boumans and Dorant 2014), which is in line with the present findings. These studies did, however, not compare different occupational types as the present study does and they only included healthcare professionals. Our results suggest that family caregiving is associated with emotional exhaustion independently of professional category.

### Strengths and limitations

The present study investigates and adds to the current state of literature regarding the so-called doubly-duty caregivers, a group on the labour market that is poorly investigated and understood. We measured human service work as time spent in direct contact with clients rather than with occupational codes—giving us the strength that the time spent in the hypothesised high-exposure work was captured. The present findings should, however, be interpreted in the light of several limitations. One of them is that the results are based on cross-sectional data, which limits conclusions about causality. With self-reported measures we cannot rule out the possibility of reverse causation, i.e., that reports of time spent in different activities could be influenced by degree of emotional exhaustion and sickness absence. Work exposures among participants in SLOSH have, however, been found to be rather stable across time and those who report working with human service have most likely been exposed to stressors associated with this type of work for a while. The associations between human service work and the outcomes found in the present study are also similar to those reported in a prospective study based on the 2012 and 2014 SLOSH data (Aronsson et al. 2019). There is furthermore in general a good agreement between self-reported and register data on sickness absence (Voss et al. 2008b). The generalizability of the results is restricted due to selective drop-out from the SLOSH study. Since drop-out from follow-ups of the study is higher among men, younger, less-educated participants and participants working in the private sector, our comparison group (those not working with human service) is less representative of the Swedish working force. Since those less represented in the sample have lower risk of emotional exhaustion and short-term sickness absence in comparison with other groups on the Swedish labour market, the differences in odds between the groups, reported in the present study, may be under-estimations (although all analyses were adjusted for gender, age and education). In addition, we most likely do not capture all individuals with mental ill-health, partly because they may be less likely to respond to follow-ups and because they may be on long-term sickness absence and thereby be excluded from the study.

## Conclusion

We did not find support for the assumption that long total hours of providing service and care for others when there is a combination of human service work and family caregiving can explain the higher risk of sickness absence or emotional exhaustion among human service workers. More research to address sickness absence among human service workers is required. Long hours in caregiving of a sick, elderly or disabled relative was found to be strongly associated with both emotional exhaustion and sickness absence, suggesting that this group may need extra support to avoid ill-health. Policies to facilitate combining family caregiving with work may be beneficial in terms of limiting the risk of emotional exhaustion for individuals irrespective of their occupation. Since caregiving patterns differ by gender we encourage researchers to explore gender differences in future studies.
